# Side Population: Its Use in the Study of Cellular Heterogeneity and as a Potential Enrichment Tool for Rare Cell Populations

**DOI:** 10.1155/2018/2472137

**Published:** 2018-12-06

**Authors:** Elize Wolmarans, Sulette Nel, Chrisna Durandt, Juanita Mellet, Michael S. Pepper

**Affiliations:** ^1^Institute for Cellular and Molecular Medicine, Department of Immunology and South African Medical Research Council Extramural Unit for Stem Cell Research and Therapy, Faculty of Health Sciences, University of Pretoria, Pretoria, South Africa; ^2^Department of Oral Pathology and Oral Biology, School of Dentistry, Faculty of Health Sciences, University of Pretoria, Pretoria, South Africa

## Abstract

There is still much to learn about the cells used for cell- and gene-based therapies in the clinical setting. Stem cells are found in virtually all tissues in the human body. As a result, cells isolated from these tissues are a heterogeneous population consisting of various subpopulations including stem cells. Several strategies have been used to isolate and define the subpopulations that constitute these heterogeneous populations, one of which is the side population (SP) assay. SP cells are identified by their ability to efflux a fluorescent dye at a rate that is greater than the main cell population. This elevated rate of dye efflux has been attributed to the expression of members of the ATP-binding cassette (ABC) transporter protein family. SP cells have been identified in various tissues. In this review, we discuss the research to date on SP cells, focussing on SP cells identified in haematopoietic stem cells, adipose-derived stromal cells, and dental pulp.

## 1. Introduction

Stem cells are increasingly being considered for their use in cell- and gene-based therapies, which constitute the most recent phase of the biotechnology revolution in medicine. Stem cells can be defined as a population of undifferentiated cells capable of proliferation and self-renewal whose differentiated progeny constitute all of the cell types of the human body [1–6].

Stem cells exist in a tightly regulated microenvironment referred to as a niche, dispersed between differentiated cells in various tissues in the body [7]. The stem cell niche does not refer to a specific location but rather to a microenvironment which provides a milieu in which the cells receive various stimuli that determine their fate or differentiation status [8]. As a result, stromal cells isolated from tissues in the human body are a heterogeneous population, consisting of subpopulations including a subpopulation of true stem cells. Various strategies have been used to isolate and define these subpopulations. In general, stem cell biology is limited by the lack of specific cell surface markers that unambiguously label the cells [9] and most investigators agree that the current “stem cell pool” consists of true (primitive) stem cells and progenitors at different stages of differentiation.

One of the methods currently used in an attempt to identify primitive stem cell subpopulations is the side population (SP) assay. The SP assay is based on the ability of cells to actively efflux a fluorescent dye [7, 10–13]. The cells are incubated for a predetermined period of time with a fluorescent dye that passively diffuses across the cell membrane. After the incubation period, the cell populations are interrogated using flow cytometry to detect and quantify the subpopulation with lower intracellular levels of the substrate, suggesting that these cells have the ability to actively efflux the fluorescent substrates at a greater rate compared to the other cells. Fluorescent dyes, such as Hoechst 33342 and Vybrant® DyeCycle™ (VDC) Violet, are usually the substrates used in the flow cytometric SP assay.

**Figure 1:**
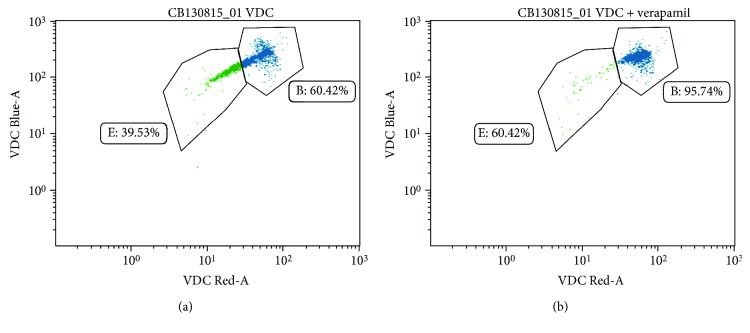
Representation of the SP in flow cytometric dot plots. (a) Dot plot showing the fluorescence pattern of hematopoietic stem and progenitor cells (HSPCs) freshly isolated from the umbilical cord blood that has been stained and incubated with the fluorescent dye VDC Violet. The main population of cells (gate B) shows greater fluorescence intensity than the cells in the tail (gate E). This tail is known as the SP and represents a subpopulation of cell with greater efflux ability than the rest of the cells. (b) Dot plot showing the disappearance of the SP tail when HSPCs are incubated with VDC Violet and the ABC transporter blocker, verapamil. Cells that were part of gate E have now moved up to join the main population of cells. This is due to the blocking effect of verapamil, which prevents the dye from being effluxed by the cells.

The assay makes use of the broad emission spectrum (ranging from around 350 nm to 650 nm) of these fluorescent substrates by measuring the changes in intracellular fluorescent emission at the optimal wavelength (460 nm; blue spectrum) as well as at the tail end of the emission spectrum (630 nm; red spectrum) [7, 10–13]. This subset of cells with decreased levels of fluorescent dye is termed the SP (Figure 1). The elevated rate of dye efflux seen in SP cells has been attributed to the expression of members of the ATP-binding cassette (ABC) transporter protein family. The Ca^2+^ channel blocker, verapamil, is often used to confirm that the decreased fluorescence intensity observed in the SP subpopulation is due to active efflux of the fluorescent substrate (Figure 1(b)). Verapamil blocks the activity of efflux proteins by reducing the membrane potential of the cells [14].

## 2. ABC Transporters

ABC transporters represent one of the largest families of membrane transport proteins and are expressed in all organisms [7, 15, 16]. The human ABC transporter family consists of 50 proteins which are divided into seven subfamilies—A to G—based on similarity in gene structure, order of the domains, and sequence homology [15–19]. Most human ABC transporters are primary active transporters, coupling the binding and hydrolysis of ATP to movement of solutes across the plasma membrane and the intracellular membranes of the Golgi apparatus, endoplasmic reticulum (ER), peroxisomes, and mitochondria [7, 15, 17]. They are known for their role in maintaining cellular homeostasis, transporting lipids and organic anions, and facilitating iron metabolism [7, 16, 18–20].

ABC transporters also play a vital role in cellular/tissue defence due to their ability to actively efflux various xenobiotics out of the cells [7, 16, 18–20]. The three most well-studied transporters known for their efflux capabilities include P-glycoprotein (P-gp; *ABCB1*), multidrug resistance-associated protein 1 (MRP1; *ABCC1*), and breast cancer resistant protein (BCRP; *ABCG2*) [11, 21–24].

## 3. Side Population and Stem Cells

Several studies have suggested that the degree of efflux activity of SP cells is inversely correlated to their maturity, with the most primitive cells (the stem/progenitor cells) having the greatest efflux activity due to the high concentration of efflux proteins on their surface [13, 25, 26]. It is hypothesized that ABC transporter expression is functionally connected to the pluripotency/multipotency of stem or progenitor cell populations and may also play an important physiological role [13, 24, 27]. It is believed that primitive, undifferentiated cells have an increased ability to efflux certain toxins, including fluorescent substrates, as a mechanism to protect themselves against potential harmful xenobiotics [28]. These theories are supported by studies comparing the expression of ABC transporters in embryonic stem cell lines and MSCs. It has been reported that different cell types have characteristic expression patterns of ABC transporters which depend on the cell's maturation state, further supporting the idea that multiple ABC transporter proteins may facilitate the pluripotency of the stem cell population [20, 27].

However, the terms “side population” and “stem cell” should not be used interchangeably, as SP cells and stem cells are not necessarily the same cells [10]. It was found that subpopulations within the SP have different immunophenotypic markers and rates of clonogenic expansion [29, 30]. It is therefore generally accepted that the SP is still heterogeneous in nature and may only contain a subpopulation of stem cells with efflux ability [10, 30]. The SP phenotype should thus be viewed as a useful enrichment strategy for the isolation of potential stem or progenitor cells from a heterogeneous population, rather than a characteristic of primitive stem cells in general. In addition, it should be taken into consideration that the dye efflux ability may not be a common property of all stem cell populations [11]. For example, Zeng and colleagues reported that undifferentiated Oct4^+^ human embryonic stem cells (H9) were Hoechst^+^ whilst the Hoechst^−^ population also contained spontaneously differentiated H9 cells [31].

The SP has been identified in various tissues (see Table 1). Here we review the research conducted to date on selected stem/stromal cell populations with a specific focus on the following:

- Characterization of SP cells
- SP cells in *in vitro* and/or *in vivo* environments
- Expression of ABC transporters and their possible roles in the SP with a focus on P-gp, MRP1, and BCRP

### 3.1. Side Population in Hematopoietic Stem Cells

In the early 1990s, several investigators observed and reported on the presence of a subpopulation (“side population”) in murine hematopoietic stem cells (mHSCs) that have the ability to actively efflux fluorescent dyes such as Hoechst 33342 and/or Rhodamine 123 [57, 58]. Goodell and colleagues improved the method used to detect these cells in 1996 [12]. The SP cells isolated from mHSCs were characterized on the basis of expression of defined mHSC cell surface markers such as Lin^−^, c-Kit (CD117)^+^, and Sca-1^+^ [12, 32, 33]. SP cells isolated from mHSCs were reported to have greater stemness properties when compared to the main population of cells. The SP cells showed increased self-renewal capacity in both cobblestone area-forming cell (CAFC) assay *in vitro* and long-term competitive repopulation experiments *in vivo* [12, 33].

Following the observation that the SP is usually lost after cells are treated with verapamil, a Ca^+^ channel and known P-gp blocker, it was assumed that the ABC transporter protein P-gp is responsible for Hoechst dye efflux [13, 24, 32]. Investigations have however revealed that even though mHSCs express various ABC transporters, BCRP is the only protein associated with the dye efflux properties of the SP phenotype [24]. It is believed that the expression of *ABCB1* and the presence of P-gp protein may not be needed for the efflux ability of these cells but may play an important physiological role in progenitor cells [21, 24].

In humans, the SP phenotype has been discovered in HSCs (hHSCs) isolated from the peripheral blood [34, 59], umbilical cord blood [35], and bone marrow [13].

Scharenberg studied the expression of the three best known efflux proteins associated with the *ABCB1*, *ABCC1*, and *ABCG2* genes in SP cells from hHSCs isolated from the bone marrow. Similar to mHSCs, *ABCG2*, which codes for BCRP, was the predominant efflux transporter gene expressed in hHSC SP cells [13].

While characterizing SP cells from hHSCs isolated from umbilical cord blood, Storms *et al.* [35] reported heterogeneity within the isolated SP cells with distinct CD34^+^ and CD34^−^ populations [35]. When comparing these two subpopulations with regard to immunophenotype and *in vitro* behaviour, they found that both Lin^−^CD34^+^ and Lin^−^CD34^−^ SP subpopulation had a similar phenotype with cells negative for CD38, Thy-1, CD33, CD45RA, and CD71 and intermediate expression levels of HLA-DR [35]. However, the Lin^−^CD34^+^ subpopulation resulted in enriched myeloid and erythroid progenitors in both short-term and 5-week long-term colony-forming unit (CFU) assays while the Lin^−^CD34^−^ SP subpopulation failed to grow in standard short-term or long-term myeloerythroid CFU assays [35]. The SP cells in hHSCs derived from peripheral blood were also reported to be CD34^+^ and CD38^−^. These SP cells displayed greater proliferative capacity and generated more clonogenic progenitors during culturing and also showed greater engraftment capabilities in xenotransplantation studies [34, 59].

### 3.2. Side Population in Adipose-Derived Stromal Cells

Adipose-derived stromal cells (ASCs) contain a subpopulation of multipotent stem/progenitor cells [2]. Over the last decade, the use of ASCs in cell-based therapies has gained increasing attention since these cells are found in abundance in adipose tissue, can easily be isolated with minor donor site morbidity, and, reportedly, have promising regenerative properties [60].

A SP has been identified in murine [29, 52, 53] and human [52, 54, 61] ASCs.

SP cells derived from murine adipose tissue have been shown to differentiate *in vitro* into myogenic, osteogenic, chondrogenic, and adipogenic lineages [29, 53]. Andersen *et al.* [29] identified SP cells in freshly isolated ASCs (known as the stromal vascular fraction (SVF)) isolated from mouse gonadal fat pads. Using immunophenotypic analysis, they identified two subpopulations within the SP: cells positive and cells negative for the common leukocyte marker, CD45 [29]. This once again highlights to the notion that the SP is not a homogenous population. The CD45^−^ SP subpopulation was enriched with cells expressing the *ABCG2* gene (along with other transcripts such as *CD31*, *CD106*, *CD133*, and endoglin) and showed greater efflux capacity when compared to the CD45^+^ SP subpopulation [29]. *ABCB1* was mainly expressed by cells in the CD45^+^ subpopulation while *ABCC1* was expressed in both subpopulations [29].

The SP cells isolated from mASCs have also been studied in various wound-healing models. Andersen *et al.* created knife-cut lesions in the *M. gastrocnemius* of NMRI mice. They showed that when CD45^−^ SP cells from ASCs (ASC-SP) were injected directly into the lesion, the CD45^−^ SP cells showed better intramuscular engraftment compared to SVF [29]. Not only did the CD45^−^ SP cells engraft better but they also differentiated into myotubes whereas only mononuclear cells were observed in the lesion area in the mice that were injected with SVF cells [29]. Ramos *et al.* studied the wound-healing abilities of SP-ASCs which were injected intradermally into NOD/SCID mice which had received a 3 mm incision through the epidermis and dermis [53]. They reported complete healing and regeneration of dermis and epidermis layers of the mice within 7 days with minimal scar formation compared to the control mice, which were injected with only PBS [53]. In 2012, Sayre and Silva [52] patented a method for using ASC-SP cells for promoting tissue regeneration. The patent states that ASC-SP cells can be isolated and transplanted via intradermal injection adjacent to the wound site, aiding in tissue regeneration in both acute and chronic wounds [52].

Little has been published on SP cells derived from human (h) ASCs with regard to their characterization or the expression of ABC transporters. The literature has mainly focussed on the therapeutic efficacy of SP cells *in vitro* and *in vivo*. Du *et al*. [61] studied the ability of cultured hASCs to differentiate into keratinocytes *in vitro*. They used a SP assay to indicate the presence of multipotent adult stem cells in ASC cultures but did not conduct further experiments to characterize the identified SP cells [61]. In their patent, Sayre and Silva [52] identified SP cells from freshly isolated mouse and human ASCs, stating that their method could be implemented with ASCs from both species. SP cells derived from hASC reportedly had an immunophenotype of Lin^−^, Sca-1^+^, CD90^+^, CD34^+/low^, CD13^+/low^, CD117^−^, and CD18^+/low^ [52]. Supronowicz *et al.* [54] studied human ASC-SP cells in tissue engineering applications; these authors used immunophenotypic markers, CD90 and CD117, in conjunction with Hoechst staining, to identify SP cells. They found that human ASC-SP cells were able to attach and proliferate on demineralized bone matrix (DBM) and to differentiate into the osteogenic lineage *in vitro* [54]. Further *in vivo* studies showed that when added to DBM grafts in a rat ectopic pouch model, hASC-SP cells enhanced bone formation [54].

### 3.3. Side Population in Dental Pulp

The dental pulp consists of highly vascular tissue that plays an important role in tooth homeostasis. This tissue is rich in natural stem and progenitor cells that play a direct role in innate healing. Cell therapies promoting the regeneration of pulp tissue have the potential to treat pulpitis or periapical disease, assuring longevity of teeth and improved quality of life [36]. In 2006, Iohara *et al.* proposed that dental pulp SP cells are enriched for stem cell properties [39]. Since then, SP cells have been identified in the dental pulp of several species including pigs [39], canines [36], and humans [9, 42].

Ishizaka and colleagues [36] isolated SP cells from canine dental pulp and compared these cells to mesenchymal stromal cell side population (MSC-SP) cells isolated from canine bone marrow and adipose tissue. Due to their gating strategy, SP cells, from all three tissues, did not express CD31 [36]. SP cells from all three tissues demonstrated CFU ability and were CD29, CD44, CD73, and CD90 positive [36]. At the molecular level, SP cells from all three tissues expressed the stem cell markers *Sox2*, *Tert*, *BMI1*, *CXCR4*, *Stat3*, and *Oct4* [36]. The regenerative capacity of SP cells isolated from dental pulp was further studied using an *in vivo* pulp regeneration model. SP cells from the three canine tissues were compared, and all were able to affect regeneration of pulp-like tissue; bone marrow CD31^−^ SP cells produced the smallest amount of regenerated tissue [36].

CD31^−^ SP cells obtained from porcine dental pulp tissue had more potent angiogenic, vasculogenic, neurogenic, and regenerative potential compared to CD31^−^ SP cells isolated from the bone marrow and adipose tissue [62]. Furthermore, condition medium from porcine pulp CD31^−^ SP cell cultures exhibited a strong stimulatory effect on angiogenesis and inhibition of apoptosis in pulp regeneration, which may be due to higher expression of *MCP1* and *CXCL14* in dental pulp SP cells [40].

In humans, SP isolated from dental pulp has increased CFU ability compared to main population cells [9]. The SP cells were able to differentiate *in vitro* into multiple cell lineages including odontoblasts/osteoblast-like cells, adipocytes, neural-like cells, and endothelial cells [9]. These SP cells expressed stem cell markers, including the *Oct4*, *BMI1*, and *Stat3* genes, at higher rates when compared to main population cells [9].

Efflux-related heterogeneity is dependent on ABC transporter proteins. Wang *et al*. [9] considered the expression of BCRP and found that only SP cells, and not main population cells, expressed BCRP. It was also found that this expression decreased over time [9]. *In situ* immunohistochemistry investigations demonstrated that BCRP is mainly expressed in endothelial cells of some microvessels and in odontoblasts at the periphery of the dental pulp [9, 63]. In earlier studies, Wang *et al*. also described BCRP expression in a small fraction of dental pulp cells (DPCs) from both deciduous (3.6 ± 0.8% DPCs expressed BCRP) and permanent teeth (2.7 ± 0.2% DPCs expressed BCRP) [41]. The expression of the *ABCG2* gene is evident in human DPCs cultured in keratinocyte growth medium [9, 64], and the rate of mRNA and protein expression has been shown to increase in human cultured DPCs under ischemic culture conditions [65]. However, in rat dental pulps, *ABCG2* mRNA expression was decreased after tooth fracture induction, possibly due to the differentiation of stem cells following this insult [66]. Even though SP cells of the dental pulp have been described as a homogenous population [39], their heterogeneous nature is becoming apparent and needs to be elucidated.

## 4. Final Comments on the Side Population Assay

The SP assay is a very sensitive but highly technical assay. Dye efflux is a dynamic process, and slight variations in tissue dissociation, cell preparation and counting, dye concentrations, staining time, temperature, the stringency of the gating strategies, and selection of the SP cells by flow cytometry can dramatically affect the viability, homogeneity, and SP cell yield [10, 11, 30]. A great deal of variation is evident when published SP data is scrutinized. Lin and Goodell [32] clearly state that the SP assay is sensitive to slight modifications leading to variable SP results from person to person and one laboratory to another [32]. Golebiewska *et al.* [11] also highlighted the importance of improving the reproducibility of SP results between laboratories and standardizing data reporting for the SP assay [11].

In summary, the SP assay should not be considered as a definite characteristic of stem cells but rather as a useful purification strategy for isolating apparently more primitive cells from the main heterogeneous population. This was well illustrated by Wilson *et al.* [67] who compared different purification strategies for the enrichment of mHSCs using single-cell analysis [67]. Their findings showed that all purification strategies, including the SP assay, resulted in a heterogeneous population of cells with differences in engraftment potential [67]. To truly understand the heterogeneity of stem or stromal cell populations, other techniques will be required.

## Figures and Tables

**Table 1:** List of tissues in which the SP has been identified.

Source	Species	References
*Hematopoietic stem/stromal cells*		
Bone marrow	Human Murine Rhesus monkey	([12]; [32]; [33]; [21]; [24]; [13])
Peripheral blood	Human	[34]
Umbilical cord blood	Human	[35]

*Embryonic stem cells*		
Immortalized cell lines	Mouse Human	([20]; [31])

*Multipotent stromal/stem cells*		
Cells derived from ESC lines	Murine	([20]; [31])
Bone marrow	Canine	[36]
Neural tissue (neural stem cells)	Human Murine	([37]; [7]; [38])
Dental pulp	Human Canine Porcine	([9]; [36]; [39]; [40]; [41]; [42])
Cardiac tissue	Human Murine	([20]; [43]; [44]; [45]; [38])
Lung tissue	Human Murine	([46]; [47]; [38])
Muscle tissue	Murine	([48]; [24]; [49]; [50]; [51]; [38])
Adipose tissue	Human Murine Rat Canine	([36]; [52]; [29]; [53]; [54])
Epidermis (keratinocytes)	Human	[55]
Endometrium	Human Murine	([30]; [56])
